# Association of life course type 2 diabetes duration with adverse cardiac structure independent of current glycaemia in the British MRC National Survey of Health and Development Birth Cohort

**DOI:** 10.1136/bmjopen-2025-113002

**Published:** 2026-10-09

**Authors:** Lamia Al Saikhan, Nishi Chaturvedi, Arjun K Ghosh, Rebecca Hardy, Alun Hughes

**Affiliations:** 1 Department of Cardiac Technology, Imam Abdulrahman Bin Faisal University, Dammam, Saudi Arabia; 2 Department of Population Science & Experimental Medicine, UCL Institute of Cardiovascular Science, London, UK; 3 Barts Heart Centre, St Bartholomew’s Hospital, London, UK; 4 Hatter Cardiovascular Institute, University College London Hospitals NHS Foundation Trust, London, UK; 5 School of Sport, Exercise, and Health Sciences, Loughborough University, Loughborough, UK

**Keywords:** Echocardiography, Diabetes Mellitus, Type 2, Cardiac Epidemiology

## Abstract

**Abstract:**

**Background:**

Type 2 diabetes mellitus (T2DM) is associated with adverse left ventricular (LV) remodelling and hypertrophy. With earlier onset of T2DM globally, understanding the impact of T2DM duration on future cardiac structure in later life is essential. This study aimed to investigate the association between T2DM duration across adulthood and cardiac structure in later life.

**Research design and methods:**

Participants from the Medical Research Council National Survey of Health and Development, (1946 British birth cohort) underwent comprehensive investigations including echocardiography at ages 60–64 years. T2DM diagnosis and age at onset were determined by self-report, physician diagnosis and/or glycated haemoglobin (HbA1c). Associations between T2DM duration and LV structure (LV mass indexed to height^1.7^ (LVMi), end-diastolic and end-systolic volumes indexed to height^1.7^, remodelling index and relative wall thickness) measured at ages 60–64 years were investigated using multivariable linear regression adjusted for potential confounders (age, sex, socioeconomic status/social class, education, body mass index, leisure-time physical activity and smoking status) and for current HbA1c.

**Results:**

Age at echocardiography was 63.25±1.10 years, and 700 (47.4%) were men. Longer T2DM duration was associated with higher LVMi independent of confounders (1.09 g/m^1.7^ (95% CI 0.36 to 1.81), per year duration; p=0.003). This association was independent of HbA1c at age 60–64 years. No associations were observed between T2DM duration and other measures of LV structure.

**Conclusions:**

Longer duration of T2DM across adulthood is associated with increased LVMi in later life, independent of current glycaemia.

**STRENGTHS AND LIMITATIONS OF THIS STUDY**

- Participants from the Medical Research Council National Survey of Health and Development (NSHD), the oldest and the longest-running birth cohort study in the UK, underwent comprehensive, repeated clinical assessments, including echocardiography, at ages 60–64 years in 2006–2010.
- Type 2 diabetes mellitus (T2DM) status was determined through self-reported physician-diagnosed diabetes or, among participants attending clinics without a prior diagnosis, a glycated haemoglobin level ≥48 mmol/mol, with previous work within NSHD cohort demonstrating good agreement between self-reported diabetes status and general practitioner medical records, supporting the validity of self-reported diagnoses in this study.
- At recruitment, the MRC NSHD cohort was broadly representative of native-born adults living in England, Scotland and Wales, but our findings may not be generalisable beyond the white British population of a similar age, to populations with higher prevalence of T2DM, or younger cohorts where levels of obesity may be higher.
- Although attrition is unavoidable in studies as long running as the MRC NSHD and clinic attendees are known to be healthier than non-attendees, this bias would be expected to attenuate, rather than exaggerate, the observed associations.

## Introduction

 The global burden of diabetes is growing rapidly with around 537 million adults (20–79 years) living with diabetes in 2021.^1^ This number is projected to increase to 643 million by 2030 and 783 million by 2045.^1^ People with diabetes are at a higher risk of developing cardiovascular diseases (CVDs),^2^ and CVD is a major cause of mortality in people with type 2 diabetes mellitus (T2DM).^3^ T2DM is associated with adverse cardiac target organ damage, including an increase in left ventricular (LV) hypertrophy and left ventricular mass index (LVMi).^4^ Increased LVMi is predictive of future CVD independent of conventional risk factors^5^; however, evidence on the impact of T2DM duration over the adult life course on LV structure, including LVMi, is limited.^6 7^ Importantly, previous studies have not investigated whether the association between diabetes duration and adverse LV structure is accounted for by poorer glycaemic control in older people with longer duration of diabetes, or whether duration of hyperglycaemia in itself is associated with cardiac structure independent of current glycaemia.

The risk of diabetes increases with age and the global age of onset of T2DM is decreasing, so it is important to understand how prolonged exposure to T2DM across adulthood affects cardiac structure later in life. This study used longitudinal data from the Medical Research Council National Survey of Health and Development (NSHD), a British birth cohort born in 1946, to examine the association between duration of T2DM during adulthood and cardiac structure in later life assessed by echocardiography at age 60–64 years in a population-based sample. We also investigated whether any observed associations were independent of current glycaemic status, since longer duration might be anticipated to be associated with poorer glycaemic control, and we wished to identify possible relationships with diabetes duration that were independent of glycaemic control.

We hypothesised that a longer duration of T2DM over the adult life course in a population-based sample would be associated with more adverse cardiac structural features in later life independent of contemporaneous glycaemia. By leveraging prospective life course data, this study offers novel insights into how the timing and chronicity of T2DM exposure may contribute to subclinical cardiovascular changes through a cumulative process beyond current glycaemia.

## Methods

### Study population

The MRC NSHD is the oldest and the longest-running birth cohort in the UK and included 5362 individuals born in a single week in March 1946 in England, Scotland and Wales. The design and characteristics of NSHD have been described previously^8 9^ and participants have undergone more than 20 follow-up visits.^8^ At age 60–64 years, 2856 eligible study participants (ie, those alive, not living outside the UK or withdrawn from the study) were invited to a comprehensive clinical examination (2006–2010) that included health, lifestyle and sociodemographic questionnaires, anthropometric measurements and detailed cardiovascular assessments.^8 9^ Of those invited, 2229 (78%) responded, of these 1690 (59.1%) attended the clinic and 539 (18.9%) had a home visit. To be included in this analysis, participants had to have an acceptable evaluation of LV mass by echocardiography and no type 1 diabetes (online supplemental material figure S1).

### Data collection

Height and weight were measured at the clinic visit and body mass index (BMI) was calculated. Two sitting blood pressure measurements from the upper right arm were recorded in clinic using an appropriately sized cuff after resting for 5 min, and the average of the two readings was calculated. Socioeconomic position was defined as the highest household occupational social class at age 53 years (Registrar General’s Social Class).^10^ Education was defined as the highest educational level achieved by age 26 years classified using the Burnham scale as follows: (1) no qualifications, (2) sub-General Certificate of Education (GCE) or sub-Burnham C, (3) GCE O level or Burnham C, (4) GCE A level or Burnham B and (5) degree or higher. Leisure-time physical activity and smoking status were collected by self-report at age 60–64 years.

Presence of T2DM was established based on questionnaire-derived self-report of doctor-diagnosed T2DM or, in those attending clinics without a preceding diagnosis, on the basis of a glycated haemoglobin (HbA1c) level≥48 mmol/mol.^11^ Previous research using the NSHD cohort has demonstrated good agreement between self-reported diabetes status and general practitioner medical records, supporting the validity of self-reported diagnoses in this study.^12^ Those with known type 1 diabetes were excluded from the analysis (n=6). HbA1c was measured at age 53 and 60–64 years by ion exchange High Performance Liquid Chromatography on a Tosoh analyser (Tosoh Bioscience, Tessenderlo, Belgium). The known age of onset of T2DM was used to calculate duration at the time of echocardiography. T2DM duration variable was then categorised into 3-year intervals for analysis; the choice of 3-year intervals was based on it being the shortest interval providing adequate numbers of observations per category and a sufficient number of interval categories. Uncategorised or 5-year interval categories were also examined as sensitivity analyses, but they made no material difference to conclusions and are therefore not presented.

### Echocardiography

1653 of 1690 participants who attended the clinic underwent echocardiography using GE Vivid I machines (General Electric, Fairfield, Connecticut, USA) performed by a trained experienced sonographer according to a strict NSHD protocol. The NSHD imaging protocol included standard echocardiographic views as follows: parasternal long-axis and short-axis, apical five-chamber, four-chamber, three-chamber and two-chamber views in addition to conventional and tissue Doppler acquisitions in the apical four-chamber view. Image optimisation was performed if required, including using second harmonic imaging. Image analysis was performed by three experienced echocardiographers blinded to patient identity using GE EchoPac software (GE Connecticut, USA) in a single core laboratory.

2D-guided M-mode LV cavity dimensions and wall thickness were measured from parasternal long-axis view from which LV relative wall thickness (RWT) was calculated.^13^ LVM was calculated according to the American Society of Echocardiography (ASE) recommendations and was indexed to body surface area (LVMi).^13^ Alternative indexation of LVM to height^1.7^ was also performed.^14 15^ LV cavity volumes and LV ejection fraction were calculated using the modified biplane Simpson’s rule.^13^

Diastolic function was evaluated in accordance with ASE recommendations, including mitral inflow diastolic filling velocities, tissue-Doppler analysis of mitral annular velocities and maximum left atrial volume calculated by the biplane method of discs at end-systole indexed to body surface area as a marker of chronically elevated LV filling pressures.^13 16^

Quality checks were performed throughout the study including blind duplicate reading reproducibility studies on a sample of studies (n=40; equal numbers of men and women and with varying image quality) to establish inter-reader and intrareader variability. The reproducibility was excellent with intraclass correlation coefficients>0.9 for all cardiac structure measurements.

The key cardiac structural measure of interest was LVMi to height^1.7^. In addition, we examined LV end-diastolic volume indexed to height^1.7^ (LVEDVi), RWT, end-systolic volume indexed to height^1.7^ (LVESVi) and LV remodelling index (LVRI) defined as LV mass-to-volume ratio measured at 60–64 years as relevant LV structural measures.

### Patient and public involvement

The NSHD study team holds annual Patient and Public Involvement and Engagement (PPIE) meetings with participants which inform research priorities. The present study was devised based on these discussions but was not co-developed with direct involvement of the PPIE panel.

### Statistical analysis

Statistical analyses were performed using Stata V.18.0 (StataCorp LLC, USA). Continuous variables are presented as mean±SD or median (IQR) as appropriate, and categorical variables are presented as counts (percentages). Associations between T2DM and cardiac structural measures at 60–64 years were assessed using multivariable linear regression models, and the β coefficient with 95% CIs is reported. Models were adjusted for prespecified potential confounders as follows: model 1: age and sex; model 2: model 1 plus occupational social class, education, BMI, leisure-time physical activity and smoking status. Confounders were chosen based on prior evidence of causal relationships to exposure and outcome. To investigate the extent to which T2DM duration influenced cardiac outcomes independent of current glycaemia we constructed model 3 which was model 2 plus adjustment for HbA1c at 60–64 years. To assess whether sex modified associations, a sex × T2DM duration term was included and p<0.05 was taken as indicative of an interaction. We found no evidence of modification of any associations by sex so results shown are from both sexes pooled. Missing values were dealt with by listwise deletion, which is valid when missingness is rare and data are missing completely at random. Details of missingness are provided in online supplemental material table S1. Inference was based on p values and 95% CIs.

## Results

### Sample characteristics

Characteristics of the study sample with echocardiography data at age 60–64 years are summarised in table 1. Mean age at echocardiography was 63.25±1.10 years, 700 (47.4%) were men and 144 (9.8%) participants had T2DM at age 60–64 years. Of those, 91 (63.2%) were identified based on self-report. Figure 1 shows the T2DM duration in the sample over the adult life course.

**Figure 1:**
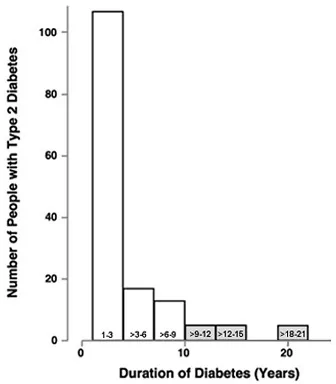
Frequency histogram showing the distribution of type 2 diabetes mellitus (T2DM) duration in the study sample. Frequency is defined as the number of people with T2DM in each duration category. For the purposes of statistical disclosure control, histograms with a frequency (number)≤10 have been shown as containing five values and displayed in grey.

**Table 1:** Characteristics of the study sample (n=1475)

Variable (at age 60–64 years unless stated otherwise)	n (%)	Mean (SD)
Age at echo, years	1475	63.3 (1.1)
Male	700 (47.4)	
Weight, kg	1465	78.2 (15.0)
Body mass index, kg/m^2^	1465	27.5 (4.6)
Socioeconomic status	1466	
Professional	124 (8.5)	
Intermediate	653 (44.6)	
Skilled (non-manual)	341 (23.3)	
Skilled (manual)	198 (13.5)	
Partly skilled	114 (7.8)	
Unskilled	35 (2.4)	
Education	1475	
None attempted	356 (24.1)	
Vocational course, proficiency only	40 (2.7)	
Sub-GCE ‘O’ level or sub-Burnham C	55 (3.7)	
GCE ‘O’ level or Burnham C	304 (20.6)	
GCE ‘A’ level or Burnham B	243 (16.5)	
Professional qualifications or Burnham A2	207 (14.0)	
First degree or graduate equivalent	179 (12.1)	
Higher degree, master’s or doctorate	17 (1.1)	
Unknown or missing	75 (5.1)	
Leisure-time physical activity	1475	
None	826 (56.5)	
1–4 times	232 (15.9)	
5 or more times	381 (26.1)	
Reported exercise but amount unknown	3 (0.2)	
Unknown	19 (1.3)	
Smoking status	1475	
Current smoker	127 (8.6)	
Ex-smoker	550 (37.3)	
Never smoker	671 (45.5)	
Unknown	128 (8.7)	
T2DM	144 (9.8)	
HbA1c, %	1383	5.81 (0.64)
T2DM duration, years	1475	2 (1, 26)*
Age of onset of T2DM, years	144	60 (37, 64)*
Previous myocardial infarction	42 (2.8)	
Systolic blood pressure, mm Hg	1471	135.7 (17.9)
Diastolic blood pressure, mm Hg	1471	77.4 (9.7)
Heart rate, beats per min	1471	68.3 (11.1)
LV mass/height^1.7^, g/m^1.7^	1475	74.7 (23.6)
LV EDV/height^1.7^, mL/m^1.7^	1475	45.5 (12.2)
LV ESV/height^1.7^, mL/m^1.7^	1475	14.3 (6.3)
LVRI	1475	1.69 (0.53)
Relative wall thickness	1475	0.43 (0.1)

* Median (range).

EDV, end diastolic volume; ESV, end systolic volume; GCE, General Certificate of Education; HbA1c, glycated haemoglobin; LV, left ventricle; LVRI, LV remodelling index; T2DM, type 2 diabetes mellitus.

### Associations between T2DM and LV structure at 60–64 years

The associations between T2DM and LVMi, LVEDVi, LVESVi, LVRI and RWT at 60–64 years are shown in table 2. T2DM was associated with greater LVMi in a minimally confounder-adjusted model (model 1) and remained independent of confounders in the fully adjusted model (model 2), although the association with T2DM was attenuated. Associations were independent of glycaemia estimated by HbA1c at 60–64 years (model 3) (table 2).

**Table 2:** Cross-sectional association between T2DM and LV structure measured at age 60–64 years

	Model 1		Model 2		Model 3	
	Coefficient (95% CI)	P value	Coefficient (95% CI)	P value	Coefficient (95% CI)	P value
	(**n=1461**)		(**n=1440**)		(**n=1350**)	
LVMi, g/m^1.7^	12.93 (9.11 to 16.67)	**<0.0001**	5.24 (1.55 to 8.92)	**0.005**	6.92 (2.11 to 11.73)	**0.005**
	(**n=1461**)		(**n=1440**)		(**n=1350**)	
LVEDVi, mL/ m^1.7^	3.45 (1.39 to 5.51)	**0.001**	0.99 (−1.09 to 3.08)	0.349	2.17 (−0.54 to 4.89)	0.116
	(**n=1458**)		(**n=1437**)		(**n=1347**)	
LVESVi, mL/ m^1.7^	1.70 (0.46 to 2.93)	**0.007**	0.88 (−0.22 to 1.99)	0.117	1.71 (0.27 to 3.16)	**0.020**
	(**n=1461**)		(**n=1440**)		(**n=1350**)	
LVRI	0.14 (0.05 to 0.23)	**0.002**	0.056 (−0.03 to 0.15)	0.247	0.04 (−0.08 to 0.16)	0.523
	(**n=1461**)		(**n=1440**)		(**n=1350**)	
RWT	0.02 (0.002 to 0.04)	**0.029**	0.008 (−0.01 to 0.03)	0.370	0.004 (−0.02 to 0.03)	0.730

Model 1: adjusted for age and sex; model 2: model 1 plus occupational social class, education, body mass index, leisure-time physical activity and smoking status; model 3: model 2 plus HbA1c at 60–64 years. P<0.05 shown in bold*.*

LV, left ventricle; LVEDVi, LV end-diastolic volume indexed to height1.7; LVESVi, LV end-systolic volume indexed to height1.7; LVMi, LV mass index; LVRI, LV remodelling index; RWT, relative wall thickness; T2DM, type 2 diabetes mellitus.

The presence of T2DM was associated with greater LVEDVi and LVRI in a minimally adjusted model (model 1), but all associations were considerably attenuated and estimates were compatible with no association when models were fully adjusted for confounders (model 2) further adjustment for glycaemia at 60–64 years (model 3) had minimal effects on strengths of associations—there was some evidence of a positive association between T2DM duration and LVESVi after adjustment for current glycaemia in model 3 (table 2).

### Associations between T2DM duration and LV structure at 60–64 years

Associations of T2DM duration with LVMi, LVEDVi, LVESVi, LVRI and RWT at 60–64 years are presented in table 3. Longer T2DM duration was associated with higher LVMi and was independent of confounders (model 2) (β coefficient=1.09 (95% CI 0.364 to 1.81, g/m^1.7^ per year; p=0.003) (table 3). When HbA1c at 60–64 years was included in models, the associations were essentially unaltered (model 3) (table 3).

**Table 3:** Association between T2DM duration and LV structure measured at age 60–64 years

	Model 1		Model 2		Model 3	
	Coefficient (95% CI)	P value	Coefficient (95% CI)	P value	Coefficient (95% CI)	P value
	(**n=1461**)		(**n=1440**)		(**n=1350**)	
LVMi, g/m^1.7^	0.93 (0.37 to 1.50)	**0.001**	1.09 (0.36 to 1.81)	**0.003**	1.14 (0.35 to 1.93)	**0.005**
	(**n=1461**)		(**n=1440**)		(**n=1350**)	
LVEDVi, mL/m^1.7^	0.23 (−0.07 to 0.53)	0.131	0.15 (−0.26 to 0.56)	0.472	0.21 (−0.24 to 0.66)	0.353
	(**n=1458**)		(**n=1437**)		(**n=1347**)	
LVESVi, mL/m^1.7^	0.09 (−0.06 to 0.25)	0.239	0.12 (−0.10 to 0.33)	0.298	0.15 (−0.09 to 0.38)	0.222
	(**n=1461**)		(**n=1440**)		(**n=1350**)	
LVRI	0.008 (−0.00 to 0.02)	0.193	0.013 (−0.01 to 0.03)	0.153	0.01 (−0.01 to 0.03)	0.243
	(**n=1461**)		(**n=1440**)		(**n=1350**)	
RWT	0.001 (−0.001 to 0.004)	0.411	0.002 (−0.001 to 0.006)	0.263	0.001 (−0.002 to 0.006)	0.386

Coefficients are per year of T2DM duration.

Model 1: adjusted for age and sex; model 2: model 1 plus occupational social class, education, body mass index, leisure-time physical activity and smoking status; model 3: model 2 plus glycated haemoglobin at 60–64 years. P<0.05 shown in bold*.*

LV, left ventricle; LVEDVi, LV end-diastolic volume indexed to height1.7; LVESVi, LV end-systolic volume indexed to height1.7; LVMi, LV mass index; LVRI, LV remodelling index; RWT, relative wall thickness; T2DM, type 2 diabetes mellitus.

There was no evidence of relationships between T2DM duration and LVEDVi, LVESV, LVRI or RWT in any models (table 3).

## Discussion

Longer duration of T2DM over adulthood in a British birth cohort was associated with higher LVMi in men and women at age 60–64 years, independent of potential confounders and current hyperglycaemia. This suggests that duration of T2DM has a cumulative adverse association with LV structure and that preventing or delaying the onset of T2DM may have benefits for future risk of CVD. Based on our observed association between T2DM duration and LVMi (a 10-year higher duration of T2DM corresponds to approximately 0.5 SD higher LVMi) this could equate to a 10–20% increase in cardiovascular event risk based on previous observations.^14^ This suggestion is consistent with a recent study showing that earlier onset of T2DM is associated with lower life expectancy.^17^ Given the current and future epidemic of diabetes and its associated high risk of CVD including coronary artery disease, heart failure and stroke, our findings may therefore have clinical and public health implications.

Previous cross-sectional studies have shown that T2DM is associated with adverse LV remodelling, including, as we observed, an increase in LVMi (ie, LV hypertrophy).^4 6 7 18 19^ In contrast, evidence from longitudinal studies regarding diabetes duration and LV structure is limited and conflicting.^6 7^ A Danish study compared patients without known heart disease and T2DM in a general population sample and found no relationship between either presence or duration of T2DM and LV mass, although interventricular septal wall thickness and RWT were higher with longer T2DM duration.^6^ In contrast, the Coronary Artery Risk Development in Young Adults study found that people aged around 50y with T2DM of duration ≤10 years had lower LV mass than those with T2DM for >10 years after adjustment for potential confounders.^7^ This latter observation is in keeping with our findings, although our sample was ~15 years older at the time of echocardiography and we provide more information about the relationship with duration by not using binary categories (early and late onset), as well as showing that the association is independent of current glycaemia. Interestingly recent evidence suggests T2DM and LV hypertrophy may have a bidirectional relationship,^20^ consistent with earlier work reporting that LV hypertrophy predicted incident T2DM in hypertensive patients.^21^ Whether such a reciprocal relationship contributes to the association between diabetes duration and LV hypertrophy merits further study.

### Limitations

At the time of recruitment, MRC NSHD was a representative sample of native-born adults living in England, Scotland and Wales, and despite attrition, the sample remains reasonably representative of the native-born UK population of comparable age.^8 22^ However, our findings may not be readily generalisable beyond the White British population of a similar age. This may be particularly relevant to communities and groups that experience more T2DM and CVD than white British or European people.^23^ Secular trends also need to be considered; data for the current analysis were collected over the years 2006–2010 from people born in Britain in 1946. More recent British cohorts have experienced more obesity and more T2DM,^24^ but the long-term health implications of these adverse trends remain to be established. Our sample had relatively low numbers of people with T2DM as would be expected in a population-based sample (for comparison the prevalence of T2DM in England has been reported to be ~9% for the age group between 55 and 64 years in 2021,^25^ compared with ~10% in this sample). This has the advantage of not including a large number of people with early onset T2DM where mechanisms may differ,^26^ but is disadvantageous in terms of statistical power and precision of estimates. The diagnosis of T2DM based on self-report is subject to recall bias, but previous work conducted in the NSHD study showed good validity of self-reported diabetes compared with general practitioner records.^12^ Timing of onset of T2DM is imprecise due to the limitations of self-report and emergence of undiagnosed T2DM between clinic or primary care visits; this imprecision is likely to result in an underestimate of the strength of association between T2DM duration and cardiac outcomes. Unmeasured or imprecisely measured confounding factors—such as lifestyle-related behaviours (eg, diet, alcohol intake, physical activity)—may have influenced both T2DM duration and cardiac structure, potentially biasing the observed associations. Assessment of an HbA1c at a single time point at age 60–64 years may not fully reflect glycaemic load and the association between T2DM duration and LVMi independent of current glycaemia may therefore have been overestimated. As expected, medication for T2DM and other conditions such as hypertension was heterogeneous in this population-based sample and we did not attempt to account for its influence. Attrition is inevitable in studies as long running as the MRC NSHD and clinic attendees are known to be healthier than non-attendees^27^; this bias might also be expected to weaken associations.

## Conclusions

Longer duration of T2DM across adult life course is associated with elevated LVMi in later life independent of current glycaemia. These findings contribute to the growing evidence base on T2DM duration and adverse cardiac remodelling.

## Supplementary material

10.1136/bmjopen-2025-113002 online supplemental file 1

## Data Availability

Data are available upon reasonable request.
