# Supplementary material for: Association of life course type 2 diabetes duration with adverse cardiac structure independent of current glycaemia in the British MRC National Survey of Health and Development Birth Cohort
Source: BMJ Open. 2026 Oct 9;16(10):e113002. doi: 10.1136/bmjopen-2025-113002 (PMC13656102; doi:10.1136/bmjopen-2025-113002)
Supplement: Supplementary file 1 [file bmjopen-2025-113002.reviewer_comments.pdf]

PEER REVIEW HISTORY

BMJ Open publishes all reviews undertaken for accepted manuscripts. Reviewers are asked to complete a checklist review form and are provided with free text boxes to elaborate on their assessment. These free text comments are reproduced below.

This paper was submitted to another journal from BMJ but declined for publication following peer review. The authors addressed the reviewers' comments and submitted the revised paper to BMJ Open. The paper was subsequently accepted for publication at BMJ Open.

ARTICLE DETAILS

Title (Provisional)

Association of Life Course Type 2 Diabetes Duration with Adverse Cardiac Structure Independent of Current Glycaemia in the British MRC National Survey of Health and Development Birth Cohort

Authors

Al Saikhan, Lamia; Chaturvedi, Nishi; Ghosh, Arjun K; Hardy, Rebecca; Hughes, Alun

---

VERSION 1 – REVIEW

**Reviewer**	**1**
**Name**	**Reviewer 1**
**Affiliation**	
**Date**	**22-Aug-2025**
**COI**	**No Competing interest**

Comments not published pending reviewer's consent

**Reviewer**	**2**
**Name**	**Reviewer 2**
**Affiliation**	
**Date**	**22-Aug-2025**
**COI**	**No Competing interest**

Comments not published pending reviewer's consent

VERSION 2 - REVIEW

**Reviewer**	**3**
**Name**	**Lee, Poay Sian Sabrina**
**Affiliation**	**National Healthcare Group Polyclinics, Clinical Research Unit**
**Date**	**21-Apr-2026**
**COI**	**None**

Thank you for the opportunity to review this manuscript. This manuscript examines the association between duration of T2DM across adulthood and cardiac structure assessed by echocardiography in later life, using data from the MRC National Survey of Health and Development. The topic is clinically relevant, given the increasing global prevalence of earlier-onset T2DM and the importance of understanding life course determinants of cardiovascular risk.

1. While the manuscript emphasises diabetes duration “across adulthood,” the results indicate that the median diabetes duration in this cohort is relatively short, with most participants diagnosed close to the time of echocardiography. This raises concerns about whether the study truly captures long-term exposure across adulthood or predominantly later-life diabetes onset.

2. The reported association between T2DM duration and LVMi (approximately 1.1 $g/m^{1.7}$ per year) is statistically robust, but its clinical relevance is not fully explored.

3. The conclusion that associations are independent of current glycaemia relies on adjustment for HbA1c measured at age 60–64 years only. While this is reasonable, HbA1c at a single time point may not fully reflect cumulative glycaemic exposure.

4. The cohort consists of White British individuals born in 1946, with relatively low obesity prevalence compared with more recent cohorts. The external validity to younger populations, ethnic minorities, and settings with higher cardiometabolic risk warrants further discussion.

5. Please clarify the extent and pattern of missing data for HbA1c and other covariates, even if missingness was low.

**Reviewer**	**4**
**Name**	**Viswanathan, Vijay**
**Affiliation**	**MV Hosp Diabetes**
**Date**	**23-Apr-2026**

COI **None**

---

1. The design and characteristics of NSHD has been described previously8,9 and participants have undergone more than 20 follow-up visits.8 At age 60-64y, 2856 eligible study participants (i.e. those alive, not living outside the UK, or withdrawn from the study) were invited to a comprehensive clinical examination (2006-2010) that included health, lifestyle and socio-demographic questionnaires, anthropometric measurements and detailed cardiovascular assessments.

- Was the sample size calculated?

2. The key cardiac structural measure of interest was LVMi to height.

- How many people give a history of MI?

3. In addition, we examined LV end-diastolic volume indexed to height1.7 (LVEDVi), relative wall thickness (RWT), end-systolic volume indexed to height1.7 (LVESVi) and LV remodelling index (LVRI) defined as LV mass-to-volume ratio measured at 60-64y as relevant LV structural measures.

- Was the regional wall abnormality noticed in some patients?

VERSION 2 - AUTHOR RESPONSE

Reviewer: 3
Dr. Poay Sian Sabrina Lee, National Healthcare Group Polyclinics

Comments to the Author:
Thank you for the opportunity to review this manuscript. This manuscript examines the association between duration of T2DM across adulthood and cardiac structure assessed by echocardiography in later life, using data from the MRC National Survey of Health and Development. The topic is clinically relevant, given the increasing global prevalence of earlier-onset T2DM and the importance of understanding life course determinants of cardiovascular risk.

1. While the manuscript emphasises diabetes duration "across adulthood," the results indicate that the median diabetes duration in this cohort is relatively short, with most participants diagnosed close to the time of echocardiography. This raises concerns about whether the study truly captures long-term exposure across adulthood or predominantly later-life diabetes onset.

Authors' response:
Thank you for your comment. In population-based samples such as the *MRC National Survey of Health and Development* T2DM is predominantly a condition that emerges in later life. For example in the UK the median age of onset of type 2 diabetes is 58.5y (women) and 58.8y (men) [Currie CJ, Currie JG, Holden SE, Morgan CL, Heywood BR, Peters JR. The descriptive epidemiology of type 2 diabetes in the United Kingdom from 2004 to 2021. 2024.

DOI:10.1101/2024.03.04.24303693.]. We now clarify that our aim was to examine this question in a population-based sample, since inclusion of disproportionate number of people with early onset T2DM could introduce bias as a result of potentially different mechanisms acting in people predisposed to early onset T2DM. We have modified the introduction and discussion to address this issue.

2. The reported association between T2DM duration and LVMi (approximately 1.1 g/m$^{1.7}$ per year) is statistically robust, but its clinical relevance is not fully explored.

Authors' response:

Thank you for your comment. We have revised the manuscript to expand on the potential clinical and public health significance of our findings, this is now considered in paragraph #1 of the discussion.

3. The conclusion that associations are independent of current glycaemia relies on adjustment for HbA1c measured at age 60–64 years only. While this is reasonable, HbA1c at a single time point may not fully reflect cumulative glycaemic exposure.

Authors' response:

Thank you for your comment, this is fair point. We have revised the manuscript to include consideration of this issue in the limitations section of the discussion.

4. The cohort consists of White British individuals born in 1946, with relatively low obesity prevalence compared with more recent cohorts. The external validity to younger populations, ethnic minorities, and settings with higher cardiometabolic risk warrants further discussion.

Authors' response:

Thank you, this too is a very valid point. We have now expanded our discussion of this issue in the context of transferability of our findings and their clinical and public health implications (Discussion).

5. Please clarify the extent and pattern of missing data for HbA1c and other covariates, even if missingness was low.

Authors' response:

This has now been provided in the supplemental material Table S1.

Reviewer: 4

Dr. Vijay Viswanathan, MV Hosp Diabetes

Comments to the Author:

1. The design and characteristics of NSHD has been described previously8,9 and participants have undergone more than 20 follow-up visits.8 At age 60-64y, 2856 eligible study participants (i.e. those alive, not living outside the UK, or withdrawn from the study) were invited to a comprehensive clinical examination (2006-2010) that included health, lifestyle and socio-demographic questionnaires, anthropometric measurements and detailed cardiovascular assessments.

- Was the sample size calculated?

Authors' response: The overall sample size was determined by the original configuration of the birth cohort. This is detailed in the references cited but we have added information on participant selection and flows to Supplemental material Figure S1. There was no sample size calculation in the sense that one might perform for a randomised clinical trial since the overall sample was set rather than drawn from a larger population, all eligible participants were studied.

2. The key cardiac structural measure of interest was LVMi to height.
- How many people give a history of MI?
Authors' response: We have now added this information to Table 1 in the revised manuscript. 42 people had a history of myocardial infarction.

3. In addition, we examined LV end-diastolic volume indexed to height1.7 (LVEDVi), relative wall thickness (RWT), end-systolic volume indexed to height1.7 (LVESVi) and LV remodelling index (LVRI) defined as LV mass-to-volume ratio measured at 60-64y as relevant LV structural measures.
- Was the regional wall abnormality noticed in some patients?
Authors' response: Unfortunately regional wall abnormalities are not available in the database.
