# Supplementary material for: Association of life course type 2 diabetes duration with adverse cardiac structure independent of current glycaemia in the British MRC National Survey of Health and Development Birth Cohort
Source: BMJ Open. 2026 Oct 9;16(10):e113002. doi: 10.1136/bmjopen-2025-113002 (PMC13656102; doi:10.1136/bmjopen-2025-113002)
Supplement: Supplementary file 2 [file bmjopen-16-10-s001.docx]

Supplemental material

| **Variable** | **Missing n** | **%** |
| --- | --- | --- |
| Age, years | 0 | 0% |
| Sex | 0 | 0% |
| Weight., Kg | 10 | 0.7% |
| Body mass index, Kg/m^2^ | 0 | 0% |
| Socioeconomic status | 9 | 0.6% |
| Education | 0 | 0.0% |
| Leisure time physical activity | 0 | 0% |
| Smoking status | 0 | 0% |
| T2DM | 0 | 0% |
| HbA1c, % | 92 | 6.2% |
| Systolic blood pressure, mmHg | 4 | 0.3% |
| Diastolic blood pressure, mmHg | 4 | 0.3% |
| Heart rate, beats/min | 4 | 0.3% |

Table S1. Missing data summary.

Figure S1. Study participant flow diagram.
